# The Ready Reckoner Model: Strengthening Healthcare Workers’ Capacity for Tuberculosis Care Through a User-Centric Digital Clinical Suite in India

**DOI:** 10.7759/cureus.113575

**Published:** 2026-07-29

**Authors:** Harsh Shah, Sandeep Rai, Jay Patel, Abhishek Sen, Rajesh Sharma

**Affiliations:** 1 Public Health Sciences, Indian Institute of Public Health, Gandhinagar, IND

**Keywords:** capacity building, digital health, health care provider, ready reckoner, tuberculosis

## Abstract

Frontline healthcare workers (HCWs) in India must navigate rapidly evolving National Tuberculosis Elimination Program (NTEP) guidelines, yet existing digital tools largely function as static repositories that are decoupled from point‑of‑care decision‑making. There is a critical need for cadre‑specific, real‑time decision support to bridge the "know-do" gap in tuberculosis (TB) care.

We developed Ni‑kshay Support to End Tuberculosis (SETU), a national‑scale digital ecosystem conceptualized as a "ready reckoner" for TB care, using a human‑centered design framework and a collaborative requirements development methodology. The platform integrates an artificial intelligence (AI)‑based chatbot, cadre‑specific multilingual clinical decision support systems (CDSS), and a knowledge hub into a single web and mobile interface. We evaluated the ecosystem using the reach, effectiveness, adoption, implementation, and maintenance framework, drawing on platform analytics, in‑app assessments, and user feedback from HCWs across India.

As of the current assessment, Ni‑kshay SETU has 46,379 active subscribers and over 1.63 million total visits, with active users in 35 of 36 states and union territories and more than 450 districts. Over 70% of users are frontline HCWs, including senior treatment supervisors, health visitors, and laboratory technicians, indicating deep penetration at the operational level. Knowledge assessments (approximately 18,900 completed) showed a statistically significant increase in mean scores from 14.49 (±6.67) at baseline to 20.46 (±7.84) at post‑intervention (p<0.001). Users reported high perceived utility for the CDSS (mean: 4.32/5) and AI chatbot (4.15/5), and 96.4% indicated they would recommend the platform to peers.

Ni‑kshay SETU operationalizes national TB guidelines into a living, digital "ready reckoner" that delivers cadre‑specific, point‑of‑care decision support at national scale. By combining human‑centered design with dynamic, cloud‑based content updates, the platform reduces cognitive load on HCWs, standardizes protocol‑compliant care, and provides a scalable blueprint for extending similar digital ecosystems to other disease programs in low‑ and middle‑income settings.

## Introduction

Digital health, or the application of digital technologies to health, has become a leading field of practice in applying routine and novel forms of information and communication technology to health needs. Digital health interventions are increasingly being used to improve the delivery of health care and to support the ongoing building of the capacity of healthcare workers (HCWs). Mobile health platforms provide a means to overcome geographical barriers and provide rapid access to evolving clinical guidelines in low- and middle-income countries [1-3]. However, current digital tools are typically dominated by e-learning repositories, program monitoring tools, or specialized diagnostic aids, with a limited integration of real-time context-specific clinical support for frontline HCWs [4,5].

This gap is relevant in the context of tuberculosis (TB) care in India, which has approximately 27% of the global TB burden [6]. The Government of India’s National Strategic Plan 2017-2025 set the target of TB elimination by 2025, which required HCWs to apply increasingly complex diagnostic and treatment protocols, including nucleic acid amplification testing and specialized regimens for drug-resistant TB [7]. The rapid evolution of National Tuberculosis Elimination Program (NTEP) guidelines has made it very difficult for traditional, instructor-led training models to keep the frontline workforce updated. There is a critical, unmet need for a "ready reckoner" -- a digital clinical companion that translates thousands of pages of complex manuals into navigable, interactive workflows [8].

Ni-kshay Support to End Tuberculosis (SETU) was conceptualized as a comprehensive digital ecosystem to address these specific functional voids and serve as a bridge between complex national guidelines and daily clinical practice. Unlike standard e-learning tools, it integrates an artificial intelligence (AI)-based chatbot, a cadre-specific multilingual decision support system, and a knowledge hub into a single mobile interface [9]. This technical report outlines the development and main features of Ni-kshay SETU and provides feedback and satisfaction levels of HCWs who have used the application. The report shares insights into the development of an integrated digital health platform to support continuous learning and access to guideline-based information in TB care.

## Technical report

**Figure 1:**
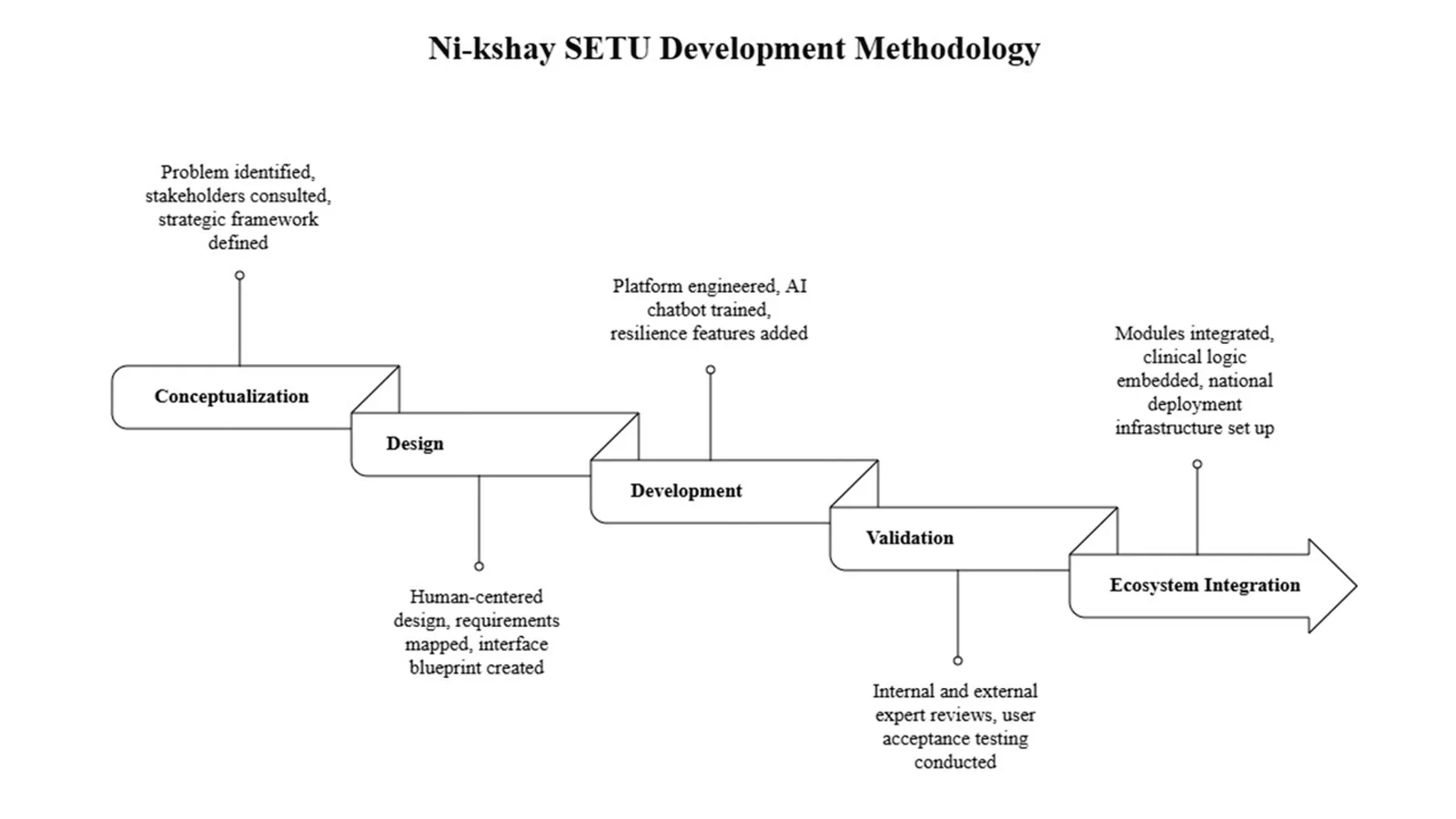
Sequential development process of the Ni-kshay SETU digital ready reckoner, illustrating the major phases (concept, design, development, validation, and deployment) and the corresponding activities undertaken at each stage AI, artificial intelligence; SETU, Support to End Tuberculosis; UAT, user acceptance testing.

Ni-kshay SETU development went through several stages before reaching its current form. A summary of this process is captured in Figure 1. The various development stages have been discussed in the subsequent parts of this report, including what was done at each stage and how it led to the development of Ni-kshay SETU.

The concept: contextual needs and strategic framework

The design of the Ni-kshay SETU ecosystem was anchored in a human-centered design (HCD) framework, moving away from a top-down information delivery model to one that prioritizes the user’s clinical environment and cognitive workflow. This process began with the development of detailed user personas, recognizing that different roles, such as medical officer (MO), an senior treatment supervisors (STS), and a laboratory technician, have fundamentally different interactions with TB data. By mapping these specific roles, the design ensured that the utility of the application was not a "one-size-fits-all" repository but a customized experience where the interface and logic changed based on the user's professional cadre [10].

A critical and unique component of the design phase was the collaborative requirements development methodology. This involved iterative consultative cycles with a diverse group of stakeholders, including WHO technical consultants, NTEP officials, and, most importantly, frontline HCWs, the ultimate consumers of the tool [11]. During these sessions, the primary design challenge was design mapping, the process of deconstructing over 2,000 pages of the NTEP clinical manuals and translating them into navigable, non-linear digital decision trees. This ensured that the application could mirror the real-world diagnostic and treatment cascades, providing specific guidance for complex scenarios such as drug-resistant TB or pediatric TB management, which are often difficult to navigate using traditional paper-based guidelines [12].

Furthermore, the design intentionally integrated nudges and a supportive supervision feature to enhance user engagement. What makes this design special is its focus on reducing the cognitive load on the provider; instead of asking the user to remember a specific drug dosage or a diagnostic algorithm, the design presents these as interactive prompts within the logic-driven workflows. This real-time decision support tool philosophy was applied to the multilingual interface as well, ensuring that the terminology used was clinically accurate yet accessible in local languages to cater to different cadres of health visitors and community workers. By focusing on these role-specific requirements and the practical constraints of field-level health care, the design phase created a blueprint for a tool that acts as a clinical companion rather than a secondary administrative burden.

The development: iterative engineering and AI optimization

The development phase of the Ni-kshay SETU ecosystem was an intensive engineering process focused on creating a seamless, cross-platform experience available through Web, Android, and iOS interfaces. The primary objective was the high-fidelity translation of the design logic into a responsive digital architecture that could withstand the demands of field use. Unlike traditional app development, which often prioritizes aesthetic over technical depth, this process is centered on the construction of a robust backend capable of processing complex clinical decision trees instantly. This allowed the platform to move beyond a simple mobile application to a comprehensive ecosystem where a user could transition between different functional modules, such as moving from a diagnostic algorithm to a specialized nutrition guide, without any loss of clinical context.

What distinguishes the development of this platform is the rigorous, multi-stage training of the AI-based chatbot. Unlike generic conversational agents, this chatbot required a specialized training regimen to ensure absolute fidelity to national protocols. This task was exceptionally rigorous, involving the ingestion and classification of thousands of verified question-and-answer pairs derived directly from official manuals. The development team used a supervised learning model where subject matter experts conducted repeated stress tests to evaluate the AI’s accuracy in interpreting varied user queries. This iterative refinement was essential to ensure the chatbot could handle cadre-specific technical inquiries, such as a laboratory technician asking about nucleic acid amplification test troubleshooting or an MO inquiring about specific drug-resistant TB regimens, with clinical precision and high speed [13].

Furthermore, the development prioritized a dynamic-update architecture. Recognizing the volatility of TB guidelines, the system was engineered to allow for real-time updates to its clinical logic via a cloud-based content management system. This ensures that when national protocols change, the point-of-care clinical companion tools on the providers' devices are updated instantly without requiring a manual app update from a store. To ensure the tool remained a functional clinical companion in low-resource settings, the development also included offline synchronization capabilities. This allows HCWs in remote areas to access the knowledge hub and previously loaded decision trees even when internet connectivity is intermittent, ensuring that evidence-based management tools are never more than a click away.

The validation: ensuring clinical integrity and accuracy

Validation was treated as a continuous quality assurance process rather than a single event, aimed at ensuring that every digital output of the ecosystem was in total alignment with the gold-standard national protocols. Because the platform provides real-time decision support, the stakes for accuracy were exceptionally high. This phase involved a rigorous two-tier verification process: internal technical validation and external expert review. The internal validation focused on code-to-clinical mapping, where medical experts within the development team tested every possible branch of the diagnostic and treatment trees to ensure that the logic-driven workflows produced the exact outcomes mandated by the latest guidelines.

The second tier of validation involved formal external reviews by a technical committee of WHO consultants, NTEP program managers, and senior academic clinicians. These experts conducted blind testing of the clinical algorithms and the AI chatbot, inputting complex patient scenarios to see if the tool's recommendations matched their expert clinical judgment. Special attention was paid to the cadre-specific outputs; for example, ensuring that the guidance provided to a health visitor regarding treatment adherence was as accurate and appropriate for their role as the drug-dosage advice provided to an MO. This rigorous scrutiny was vital to ensure that the tool could function as a trusted clinical companion in diverse field conditions.

Following the expert review, the platform underwent a final user acceptance testing phase. This involved a pilot group of frontline HCWs who used the platform in a simulated clinical environment. Their feedback was used to identify any remaining friction points in the interface or any ambiguities in the chatbot’s responses. This step ensured that the tool was not only clinically accurate but also practically usable for different professional cadres. By the end of the validation phase, the ecosystem had been verified as a high-fidelity digital translation of the national manuals, ready for large-scale implementation as a reliable on-demand knowledge repository.

The Ni-kshay SETU ecosystem: a multi-dimensional clinical suite

The culmination of the design and development process resulted in a comprehensive digital ecosystem that integrates various functional modules into a single, cohesive interface. Rather than functioning as standalone utilities, these features are interconnected to support the HCW throughout the entire spectrum of patient care. The final architecture was specifically engineered to move beyond the limitations of static applications, providing a dynamic environment where clinical logic, peer-to-peer communication, and patient-centric tools coexist to improve the quality of care.

The core of the system is the AI chatbot, a sophisticated, voice-assisted interface trained to interpret natural language queries and provide immediate, guideline-based responses. This serves as an "always-on" assistant that can clarify complex clinical protocols during a patient encounter without requiring the user to navigate through manual menus. Complementing this is the knowledge hub, an on-demand repository of role-specific, multilingual learning materials and instructional videos that facilitate continuous professional development. To ensure that this knowledge is effectively retained, the knowledge quiz module provides a gamified assessment environment where users can test their expertise through cadre-specific evaluations, offering instant feedback and tracking personal progress.

For active clinical management, the platform features a highly specialized Treatment and Diagnostic Cascade module and a clinical decision support system (CDSS). These components translate complex national algorithms into interactive workflows that guide the HCW through appropriate diagnostic pathways and treatment regimens based on the specific risk profile of the patient. The CDSS acts as the intellectual engine of the application, ensuring that even the most complex cases follow evidence-based protocols. This is further supported by the Query2CoE feature, which provides a direct channel for providers to seek expert clarification from their respective center of excellence for TB Care on challenging cases. Additionally, the TB Nutrition Guide offers cadre-specific counseling tools to address the critical link between malnutrition and TB recovery, providing actionable dietary advice that can be shared directly with patients.

The ecosystem also extends its utility into the community through the Screening Tool, which uses a systematic questionnaire to identify presumptive cases, and the Referral Facilities module, a geospatial mapping tool that identifies the nearest of over 75,000 diagnostic and treatment centers. To ensure continuity of care after the patient leaves the clinic, the TB Care Message Hub was integrated to facilitate supportive supervision and stage-specific communication. Finally, the inclusion of Differentiated TB Care logic ensures that patients at higher risk of poor outcomes are identified early, allowing for a more personalized and intensive management approach. Together, these features transform the platform from a simple information tool into a robust clinical companion that supports the HCW at every critical juncture of the TB care journey.

Evaluation framework: the reach, effectiveness, adoption, implementation, and maintenance model

To systematically assess the impact and performance of the Ni-kshay SETU ecosystem, the study employed the reach, effectiveness, adoption, implementation, and maintenance (RE-AIM) framework [14]. This multi-dimensional model was selected to move beyond simple usage statistics and provide a holistic view of the platform’s public health value. By using this framework, the evaluation could move past vanity metrics such as app downloads and instead focus on the actual utility of the tool in the hands of HCWs. Within the RE‑AIM framework, reach was defined as the absolute number, proportion, and geographic distribution of active Ni‑kshay SETU users across India. Effectiveness was assessed through changes in in‑app knowledge assessment scores. Adoption captured the number and diversity of cadres and districts integrating the tool into routine practice. Implementation focused on actual use of core modules (AI chatbot, CDSS, and treatment cascades) relative to their intended design, and maintenance examined patterns of sustained use and the platform’s ability to remain aligned with evolving NTEP guidance over time.

Reach: National Penetration and Grassroots Engagement

The reach of the Ni-kshay SETU platform highlights its effectiveness as a national-scale capacity-building tool. As of the current assessment, the ecosystem has secured 45,886 active subscribers (month) and recorded over 1.63 million total visits, reflecting a high level of recurring engagement rather than one-time downloads. A critical success of the platform’s dissemination is its near-universal geographical coverage, with active users identified in 35 out of 36 states and union territories across India, covering more than 450 districts.

Furthermore, the data indicate that the platform achieved its goal of universal access across the healthcare hierarchy. Over 70% of the total user base consists of frontline HCWs, including STS, health visitors, and lab technicians. The remaining 30% comprises MOs, medical interns, state and national consultants, program managers, and senior TB officials. This confirms that the platform’s design successfully overcame barriers to digital entry for the most vital cadres in the TB care cascade, ensuring that the latest clinical guidelines are accessible at the primary point of care.

Effectiveness: Quantifiable Knowledge and Confidence Gains

The effectiveness of the intervention was assessed through knowledge gain using data obtained from the knowledge quiz and assessment modules embedded within the Ni-kshay SETU platform. The knowledge assessment module was designed to record only completed assessments. Incomplete assessments or instances where participants did not complete either the pre- or post-intervention assessment were not captured by the application and, therefore, were not included in the analysis. Consequently, all participants with complete paired pre- and post-intervention assessments were included in the analysis.

A total of 18,900 paired pre- and post-intervention assessments completed by the same HCWs were included in the analysis. The mean knowledge score increased from 14.49 (±6.67) at baseline to 20.46 (±7.84) following the intervention. Comparison of paired pre- and post-intervention scores using a paired t-test demonstrated a statistically significant improvement in knowledge (p<0.001). Beyond pure knowledge acquisition, the availability of the CDSS and the AI chatbot (which has processed thousands of unique clinical queries) has directly contributed to increased provider confidence. This is particularly evident in the management of complex cases, such as drug-resistant TB and pediatric TB, where the tool acts as a "digital companion" to ensure every decision aligns with the gold-standard national protocols.

Adoption: Systemic Integration Across Professional Cadres

Adoption metrics reflect the extent to which the platform has been integrated into the professional lives of diverse stakeholders. The ecosystem’s adoption was not limited to individual learning; it was systematically integrated into the programmatic workflow of over 450 districts. The high adoption rate among both public and private sector providers suggests that the tool effectively addressed the information void identified in the conceptual phase. By providing cadre-specific interfaces, the platform ensured that an MO looking for drug regimens and a laboratory technician looking for diagnostic protocols both found the tool equally indispensable to their specific roles.

Implementation: Technical Fidelity and Navigational Ease

Implementation success was evaluated by the fidelity with which the platform’s features were used relative to their design logic. The high frequency of interaction with the AI chatbot and the Treatment and Diagnostic Cascade modules suggests that users used the platform as a real-time clinical assistant rather than a static library. The technical implementation remained stable across Web, Android, and iOS interfaces, maintaining high performance and speed. This technical reliability ensured that the ready-reckoner philosophy was realized, allowing HCWs to navigate complex clinical pathways in high-pressure field environments without technical friction.

Maintenance: Long-Term Sustainability and Policy Responsiveness

The maintenance dimension focuses on the platform’s ability to remain a permanent and evolving asset within the national health system. The cloud-based, dynamic-update architecture ensures the platform’s longevity; as NTEP guidelines evolve, the logic within the CDSS and AI chatbot is updated centrally and pushed to all users instantly. This structural flexibility, combined with high user retention rates, positions Ni-kshay SETU as a sustainable public health asset. It serves not just as a temporary intervention but as a scalable infrastructure that can be adapted for other disease-specific programs, ensuring long-term impact on the quality of healthcare delivery.

User perception and experience: a post-implementation feedback analysis

The transition from technical validation to large-scale implementation necessitated a deep understanding of user experience. We believed it was fundamental to establish a robust feedback loop, as the true measure of a digital health tool lies not just in its algorithmic accuracy but in its daily utility for the end-user. By systematically capturing the voices of the HCWs, we aimed to move beyond developer-centric assumptions and refine the ecosystem into a truly user-centric clinical companion that simplifies, rather than complicates, the TB care journey.

Quantitative Performance

To assess user perceptions and experiences with the Ni-kshay SETU ecosystem, user engagement data from the Ni-kshay SETU ecosystem were analyzed to identify active users. For the purpose of this assessment, an active user was defined as an HCW who had accessed the Ni-kshay SETU application on multiple occasions and engaged with its core features, including the knowledge hub, chatbot, knowledge assessment tool, and/or cadre-specific decision-support tools. Users demonstrating the highest levels of engagement based on these activity indicators were invited to participate in the user feedback survey. Participation was voluntary, and only HCWs who provided informed consent and completed the pretested, semi-structured questionnaire were included in the analysis.

A total of 196 HCWs provided complete responses and constituted the final analytic sample. Table 1 summarizes the quantitative performance of the Ni-kshay SETU ecosystem based on primary feedback variables (rated on a 5-point Likert scale) collected from 196 HCWs using a pretested semi-structured questionnaire administered through Google Forms. The completed questionnaire responses were exported and analyzed using descriptive statistics in Jamovi (version 2.6; the Jamovi Project, Sydney, Australia), and the results are presented as mean scores for each feedback domain. Overall user satisfaction, ease of navigation, technical relevance, and feature‑specific utility all scored above 4.1 on a 5‑point Likert scale, underscoring both perceived clinical relevance and usability of the ecosystem.

**Table 1:** Performance of the Ni-kshay SETU ecosystem AI, artificial intelligence; CDSS, clinical decision support system; SETU, Support to End Tuberculosis.

Performance metric	Mean score (out of 5)	Feature utility	Mean score (out of 5)
Overall user satisfaction	4.29	Knowledge connect	4.38
Ease of navigation	4.29	Decision support (CDSS)	4.32
Technical relevance	4.26	Knowledge quiz	4.29
Feature placement	4.26	Screening tool	4.20
Shorthand interpretation	4.19	Treatment cascade	4.18
App speed/performance	4.15	SETU AI chatbot	4.15

The analysis highlights that the HCWs found the knowledge hub to be the most impactful feature for their professional growth, while the CDSS provided the necessary logic-driven support for managing complex drug-resistant cases. The high score for ease of navigation is a testament to the HCD approach, ensuring that even under the pressures of a busy clinic, the HCW can find critical information without technical friction.

Qualitative Reflections

Beyond the numbers, the qualitative feedback provided a deeper look into the perceived value of the platform. The following testimonials from various cadres of HCWs illustrate the ecosystem's role as both a learning tool and a clinical assistant: (a) “It was very helpful in understanding TB protocols and managing patients more effectively.” (b) “A very useful and informative app for our day-to-day management of TB cases.” (c) “Great initiative; it makes the complex NTEP guidelines much more accessible and easier to follow at the field level.” (d) “The chatbot is excellent for quick clarifications when we don't have time to look through manual books.”

The high recommendation rate of 96.4% further reinforces these testimonials, suggesting that the platform has successfully fostered a sense of professional trust. By interpreting medical abbreviations and providing a ready reckoner for diagnostic cascades, the ecosystem has managed to reduce the cognitive load on HCWs, allowing them to focus more on patient care and less on administrative or protocol-related uncertainty.

## Discussion

This study was conducted to explore the potential of Ni-kshay SETU, a national-level, cadre-based digital ecosystem, to enhance the knowledge level of health workers and its scalability as a point-of-care tool for implementing guidelines for TB. RE-AIM framework analysis revealed a large national reach (46,379 active subscribers in 35 out of 36 states and over 450 districts), significant gain in the knowledge scores of the user base completing the paired assessment (mean difference in score from 14.49 to 20.46, p<0.001), and high utility for the core decision-making support functions of the platform. Collectively, the above observations suggest that the platform succeeded in making tangible gains in terms of reach and effectiveness under the RE-AIM framework.

The extent of the gains in knowledge in the current study is comparable to other studies conducted using similar intervention strategies for mobile learning and decision support systems for frontline health workers in low- and middle-income countries. For instance, a mobile-based mLearning application focusing on postpartum hemorrhage and neonatal resuscitation in Rwanda increased knowledge by 19.1 points out of 100 in nurses and midwives, while a mobile antenatal-care decision-support application in Nigeria was able to increase a quality-of-care score from 13.3 to 17.2 after the introduction [15,16]. The consistency in the magnitude of the gains between the diseases and their respective mobile learning and decision support interventions (TB, obstetric, and maternal health) implies that the gains are probably a result of common factors in the three interventions and not the specific content of each, and these include the structured approach of delivering the content guided by algorithms and assessment of the knowledge gained. The findings are consistent with existing research showing that mobile and digital learning methods enhance the skills of HCWs [17].

The finding that more than 70% of active users were frontline cadres (senior treatment supervisors, health visitors, and laboratory technicians) is in line with existing evidence that mHealth tools can support task-sharing by extending access to technical knowledge beyond specialist cadres [18]. A similar pattern of frontline uptake has been reported for community health workers using a mobile decision-support tool for hypertension management in Guatemala and for antenatal-care providers in Nigeria [19]. These findings support the inference that a cadre-specific interface, rather than a generic repository, is a common design feature associated with high frontline adoption. A key distinction, however, is that the Guatemala and Nigeria programs were operated within defined pilot cohorts with structured field supervision, whereas Ni-kshay SETU was adopted voluntarily at large scale without an equivalent supervisory structure. This suggests that a well-designed, cadre-specific digital interface may substitute, at least partially, for close in-person supervision in promoting adoption, although the present study did not directly measure whether this translated into equivalent gains in clinical performance.

A high rating in relation to the perceived usefulness of both the CDSS (4.32/5) and AI chatbot (4.15/5) is also corroborated by the literature that has investigated similar systems, noting their potential to reduce cognitive burden and promote adherence to the standardized clinical pathway. Similarly, adherence to the protocol for neonate care was shown to improve after implementing a mobile decision-making support system in Ghana [20]. The rating of the chatbot in this case is relatively similar to that of the CDSS, which shows that a conversation interface is useful when it comes to the resolution of context-based questions, alongside the algorithmic-based routes, and is in line with what has been observed in a decision support tool used by community health workers in Guatemala, in which the user's trust and agreement with treatment recommendations have progressively improved with greater experience of the tool [19]. However, the current research differed from the cases in this study. There was no observation of adherence to protocol and prescription agreement but only an evaluation of perceived utility and knowledge scores.

The results should be interpreted with some limitations in mind. Selection bias may have been introduced in the knowledge-gain analysis, as we only included users who completed paired pre- and post-intervention assessments. Similarly, the feedback survey was completed by highly engaged users and may therefore have overrepresented more favorable perceptions of the platform. The lack of a comparison group in the uncontrolled pre-post design also limits our ability to attribute the observed knowledge gains solely to platform use, as concurrent training activities or broader increases in awareness of NTEP guidelines may have contributed. Importantly, the study measured platform reach, knowledge gained, and perceived utility but not change in clinical practice, adherence to treatment guidelines, or patient-level outcomes. Thus, while the findings confirm the feasibility and acceptability of a cadre-specific, AI-supported digital platform to expand access to TB-related clinical knowledge, longitudinal and controlled studies are needed to see whether sustained use of the platform translates into improved guideline adherence, quality of care, and patient outcomes.

## Conclusions

The use of Ni-kshay SETU was common among the HCWs, and they received it very positively. The notable increase in knowledge scores shows that the platform helped HCWs comprehend TB guidelines. By integrating cadre-based content with decision support at the point of care, Ni-kshay SETU can enable HCWs to understand the complicated TB guidelines. Further studies are needed to determine whether continued use of the platform improves clinical practice and patient outcomes.
